# Around the Hospitals

**Published:** 1905-08-26

**Authors:** 


### AROUND THE HOSPITALS.

### MATTRESS AIRING ROOM AT MIDDLESEX.

As the airing of clothes, bedding, etc., is part of the process included in laundry work, the arrangement of the mattress room at Middlesex hospital will be of interest. The mattresses are placed on separate galvanised iron grids, one above another, with a space between, so that air can pass freely all round them, and get to all parts. Three hundred mattresses are stored in the room, and they are kept dry by the following arrangement. There are two windows, each of which has a ventilator, and there is a fan on the opposite wall, between the two windows, the fan drawing the air from the windows through the spaces between the mattresses. At each window is one of Teale's ventilators and oil-bath cleaners, consisting of short zinc tubes leading to a bath of oil, and thence into the chamber. Any dirt that may be in the air being heavier than the oil, falls to the bottom of the oil bath, so that the air is presented to the room cleaned.

The mattress room is heated by hot pipes passing through the room, independently of the ventilation, the air being drawn over the pipes by the action of the fan. In the new hospital at Belfast, the writer understands that good effects have been obtained by passing more air than is usually allowed through the drying closets, and allowing the air to pass from them to the washhouse itself, the air being taken originally from the ironing room. The steamy atmosphere of the washing room is always a matter of some anxiety, and it is claimed that, at Belfast, by not saturating the air that passes through the drying closets, and allowing it to escape into the washhouse, with a capacity for moisture unsatisfied, the steamy atmosphere is got rid of, to a large extent. It will be noticed that the course of the air in the laundry at Belfast is very similar to that at the London Hospital.

### CHARING CROSS HOSPITAL.

#### The New Surgical Block.

At the top are two operating theatres, lecture theatre, and clinical laboratories; the new electrical and out-patient departments, already described in these columns, are on the basement and sub-basement, whilst the intervening floors are devoted to provision for 90 beds in six wards, three on each floor. Cross-ventilation has been secured, and the wards are lofty and light. The cement floors are covered with linoleum. The lockers, made to the design of one of the staff, are of teak, and run under the Lawson Tait bedsteads. The bed-tables are also of teak, and have a book-rest in the middle which can be raised or lowered at will. Heating is effected by means of central stoves. At the end of each ward is a sanitary annex, and a gallery leading to the Nurses' Home for use in case of fire. Between the wards are convenient kitchens. In the operating theatres, which are tiled and walled with white marble, the plan of interposing a glass screen between the floor of the theatre and the students' gallery is about to be tried. The theatres are ventilated by filtered air brought in from outside, the windows being kept closed.

